# Metabolic Alterations in Different Stages of Hypertension in an Apparently Healthy Nigerian Population

**DOI:** 10.1155/2013/351357

**Published:** 2013-11-25

**Authors:** M. A. Charles-Davies, A. A. Fasanmade, J. A. Olaniyi, O. E. Oyewole, M. O. Owolabi, J. R. Adebusuyi, O. Hassan, M. T. Ajobo, M. O. Ebesunun, K. Adigun, K. S. Akinlade, U. A. Fabian, O. O. Popoola, S. K. Rahamon, W. Okunbolade, M. A. Ogunlakin, O. G. Arinola, E. O Agbedana

**Affiliations:** ^1^Department of Chemical Pathology, College of Medicine, University of Ibadan, Ibadan 200284, Nigeria; ^2^Department of Medicine, College of Medicine, University of Ibadan, Ibadan 200284, Nigeria; ^3^Department of Haematology, College of Medicine, University of Ibadan, Ibadan 200284, Nigeria; ^4^Department of Health Promotion and Education, College of Medicine, University of Ibadan, Ibadan 200284, Nigeria; ^5^Medical Social Services Department, University College Hospital, Ibadan 200212, Nigeria; ^6^Dietetics Department, University College Hospital, Ibadan 200212, Nigeria; ^7^Department of Chemical Pathology, College of Health Sciences, Olabisi Onabanjo University, Ago-Iwoye 120005, Nigeria; ^8^General Out Patient Unit, University College Hospital, Ibadan 200212, Nigeria

## Abstract

Metabolic syndrome (MS) amplifies hypertension (HTN) associated with increased risk of cardiovascular disease (CVD). MS components and other CVD risk measures were investigated in different stages of hypertension. 534 apparently healthy Nigerian traders aged 18–105 years were participants of a cohort study. The International Diabetes Federation (2005) and the National High Blood Pressure Education Program Coordinating Committee criteria were used for MS and HTN classifications, respectively. Anthropometric indices were obtained by standard methods. Levels of fasting plasma glucose (FPG), total cholesterol (TC), triglyceride (TG), and high-density lipoprotein cholesterol (HDLC) were determined by enzymatic methods, while low-density lipoprotein cholesterol (LDLC) was calculated. Data analysed statistically were significant at *P* < 0.05. 143 (26.8%), 197 (36.9%), and 194 (36.3%) of the traders had normotension, pre-HTN and HTN (stages 1 and 2), respectively. All indices tested except HDLC were significantly different among BP groups (*P* < 0.05). Waist to hip (WHR) and waist to height (WHT) ratios were significantly different between HTN groups (*P* < 0.05). HTN was associated with MS and female gender (*P* < 0.05). Metabolic alterations and significant HTN were observed. Treatment of the individual components of the syndrome and improvement of modifiable metabolic factors may be necessary to reduce MS and high BP.

## 1. Introduction

Hypertension (HTN) is an important public health issue worldwide and it is associated with increased risk of cardiovascular and renal diseases. It is defined as blood pressure (BP) ≥ 140-systolic (SBP) and/or ≥90 mmHg-diastolic (DBP) or being on drug therapy [1]. BP (SBP and/or DBP) for adults aged ≥18 years has been classified as normal (<120 and <80 mmHg), pre-HTN (120–139 or 80–90 mmHg), stage 1 HTN (140–159 or 90–99 mmHg), and stage 2 HTN (≥160 or ≥100 mmHg) [2]. HTN is more prevalent in Africa and associated with more complications in Blacks. It is the most prevalent cause of end-stage renal disease in West Africa and an important cardiovascular risk factor worldwide [1, 3, 4]. Ulasi et al. [1] reported an alarming prevalence of HTN in 42.2% of market workers with majority of them not aware of the disease. The high prevalence of HTN was attributed to their sedentary lifestyle, consumption of salt-laden fast food, and obesity.

HTN is one of the components of metabolic syndrome (MS), a cluster of risk factors that includes obesity, insulin resistance, HTN, and dyslipidemia unequivocally linked to an increased risk of developing type 2 diabetes (DM) and cardiovascular disease (CVD) [5]. MS and its co-morbidities hyperglycemia, abdominal obesity, and hypertriglyceridemia were found to be higher in hypertensives compared to the general population [6]. MS is prevalent among traders in Nigeria and has been associated with obesity and gender [7]. The MS amplifies cardiovascular risk associated with high BP in hypertensives, independent of the effect of several traditional cardiovascular risk factors [8]. The double burden of communicable and noncommunicable diseases are well known in Africa with economic implications [1, 3]. Early detection and management of HTN-related metabolic derangements may prevent pandemic of morbidity and mortality associated with the disease. The association of different stages of HTN and various measures of cardiovascular risk has not been adequately investigated among Nigerians. Components of the MS and other CVD risk measures were thus investigated in different stages of HTN among apparently healthy Nigerian traders.

## 2. Subjects and Methods

### 2.1. Study Design

The study was a cohort study conducted over a period of 6 months and ethical approval was obtained from the Joint Ethical Committee of the University of Ibadan/University College Hospital, Ibadan, Nigeria (UI/UCH).

### 2.2. Participants

534 (170 males and 364 females) apparently healthy traders from a local market in Bodija, Ibadan, aged 18–105 years participated in the study. In collaboration with leaders of their market association who were adequately informed about the study in their local language, Yoruba, recruitment of the participants was done by consecutive selection of all apparently healthy traders without DM (from their pretest questionnaire) that gave informed consent. Selection was not based on age, gender, or HTN. They were part of a cohort study on Risk Assessment of DM in Individuals with MS in Ibadan, South-West, Nigeria, conducted in the Department of Chemical Pathology, College of Medicine, UI.

### 2.3. Classes of HTN

Participants were classified into different BP (SBP and/or DSP) groups-normal (BP: <120 and <80 mmHg), pre-HTN (BP: 120–139 or 80–89 mmHg), stage 1 HTN (BP: 140–159 or 90–99 mmHg), and stage 2 HTN (BP: ≥160 or ≥100 mmHg) [2]. 

### 2.4. Diagnosis of MS

MS was made using the International Diabetes Federation (IDF) diagnostic criteria [9]. The criteria include central obesity (WC) (male: ≥94 cm, female: ≥80 cm) and any two of raised triglycerides (TG): ≥150 mg/dL (1.7 mmol/L), reduced high-density lipoprotein cholesterol (HDLC) (males: <40 mg/dL, females: < 50 mg/dL, raised BP (≥130/≥85 mmHg), or raised fasting plasma glucose (FPG) (≥100 mg/dL). 

### 2.5. Components of MS

These were parameters contained in the IDF for MS-WC, TG, HDLC, BP, and FPG [9]. These were measured by standard methods described elsewhere [7, 10].

### 2.6. Measures of Adiposity and Lipids

Adiposity measures included body weight, height, body mass index (BMI), hip circumference (HC), waist hip ratio (WHR), waist height ratio (WHT), and percentage body fat (PBF). Lipids (other than HDLC and TG-components of MS) were total cholesterol (TC) and low-density lipoprotein cholesterol (LDLC). Methods described by Umoh et al. [10] and Charles-Davies et al. [7] were used for these measures.

### 2.7. Sample Collection

6 mL of venous blood sample was aseptically obtained by venipuncture from the participants after an overnight fast (10–14 h). 4 mL was dispensed into potassium ethylene diamine tetra acetic acid (K3EDTA) tube for the determination of lipid profile (TC, TG, and HDLC). 2 mL was dispensed into fluoride oxalate tube for FPG estimation. All samples were centrifuged at 500 g for 5 min after which plasma/serum were aspirated in small aliquots into clean vials and stored at −20°C until analyses were done.

### 2.8. Statistical Analysis

Data were analyzed using the Statistical Package for Social Sciences (SPSS) software 15.0 version. Post hoc tests were used for comparison of quantitative variables. Chi-square test (*χ* ^2^) was used to find relationship between two qualitative variables. Two-tailed independent *t*-test of significance at 95% confidence limit with *P* < 0.05 was considered significant for the variables. 

## 3. Results and Discussion

HTN is frequently undiagnosed until relatively late in its course leading to kidney damage and heart failure [11]. In this study, 143 (26.8%) of the traders had normotension while 194 (36.3%) of them had stages 1 and 2 HTN. This is rather alarming for a group of apparently healthy individuals. Our findings corroborate those of Ulasi et al. [1] in a similar group studied. 

 HTN is a known component of MS, a syndrome that increases the risk of developing CVD and DM.  Table 1 shows significant associations of BP with MS and female gender (*P* < 0.05). MS represents a strong and independent risk factor for future CVD in patients with HTN, over and above the prognostic information provided by all other traditional cardiovascular risk markers [8, 12]. Among the participants who had MS, 10 (5.6%) had normotension, 44 (24.9%) had pre-HTN, while 123 (69.5%) had stages 1 and 2 HTN. On the other hand, among the participants with non-MS, 133 (37.3%) had normotension, 153 (42.9%) had pre-HTN, while 71 (19.9%) had stages 1 and 2 HTN. Bulhoes and Araujo [13] reported a prevalence of 0.8 to 35.3% as a range of metabolic abnormalities associated with HTN in control and HTN groups. The male : female distributions among the blood pressure groups in our study were normotension—31.8% versus 24.5%, pre-HTN—47.1% versus 32.1%, and HTN (stages 1 and 2) 21.1% versus 43.4%, respectively. Associations of MS with obesity and female gender were previously observed by our team [7].

 HTN is associated with the laboratory and anthropometric findings linked to MS. Central obesity and insulin resistance recognised as the main factors involved in the pathophysiology of the MS have been postulated to contribute to elevated blood pressure, which further promotes vascular damage in cardiac, renal, and brain tissues [11].  Table 2 shows comparison of MS components (BP-SBP and DBP, WC, FPG, TG) between BP groups (using post hoc tests). There were significant increases in levels of BP from normal to stage 2 HTN (*P* < 0.001). Significant increases in WC (a measure of central obesity/visceral fat) from normal to stage 2 HTN were also observed between all groups (*P* < 0.003) except in the comparison between stage 1 and stage 2 HTN (*P* > 0.05). Visceral fat is a metabolically active organ that is strongly related to insulin sensitivity. Visceral adipose tissue secretes leptin, an adipocytokine associated with the processes of inflammation, endothelial dysfunction, HTN, and atherogenesis [11, 14, 15]. Obesity, especially visceral adipose tissue accumulation, increases the risk of developing DM and may be linked to HTN through sympathetic nervous system overactivation [7, 11, 16]. Results of our previous study showed that increases in leptin levels in individuals with and without MS and DM might reflect adiposity as well as a compensatory mechanism for maintenance of weight/fat loss and BP [16]. 

Insulin resistance and the resulting hyperinsulinemia induce BP elevation by the activation of the sympathetic nervous system and renin-angiotensin-aldosterone system with consequential sodium retention and volume expansion, endothelial dysfunction, and alteration in renal function [11]. Syed et al. [17] observed impaired and high glucose levels in pre-HTN, while Ulasi et al. [6] observed hyperglycemia in the HTN group in their study. Contrarily, in this present study, significant increases in the mean FPG concentrations from normotension and stage 1 HTN to stage 2 HTN were observed (*P* < 0.05, Table 2), although these levels were within the normal reference range.

The mean TG levels in this present study increased significantly from normotension to stages 1 and 2 HTN as well pre-HTN to stage 2 HTN (*P* < 0.05). Again, these levels in all HTN groups were within normal reference range, a finding contrary to an earlier report of significant hypertriglyceridemia in HTN in a similar population in Nigeria [6]. The mean (s.d) levels of HDLC in normotension, pre-HTN, stage 1 HTN, stage 2 HTN and all participants were 1.1 (0.3), 1.1 (0.4), 1.1 (0.5), 1.1 (0.4), and 1.1 (0.4) mmol/L, respectively. Comparisons of mean HDLC levels among all BP groups did not show significant differences (*P* > 0.05). Moreover, HDLC levels were generally low-normal levels in the apparently healthy traders. These findings suggest that the contribution of HDLC (a MS component) to the high prevalence of HTN in this present study may be limited.

Table 3 shows comparisons of means of non-MS components among BP groups using post hoc tests. The mean height, body weight, BMI, HC, WHT, WHR, PBF, TC, and LDLC showed significant differences among BP groups (*P* < 0.05). The mean WHR and WHT were significantly different between all BP groups (*P* < 0.05). WHR is positively and independently related to the occurrence of arterial HTN [17]. WHT (an improved index over WC) is a simple and practical index for assessing central fat distribution and metabolic risk in men and women [18].

The mean height, body weight, BMI, and HC were significantly different (*P* < 0.05) among all BP groups except those between stages 1 and 2 HTN (*P* > 0.05). There were significant reductions in height from untreatable groups (normotension and pre-HTP) to treatable groups (stages 1 and 2 HTN). Significant differences were observed in the comparison of PBF between normotension and HTN (stages 1 and 2) groups as well as between pre-HTN and HTN (stages 1 and 2) groups (*P* < 0.05). There were also significant increases in TC and its lipoprotein-LDLC from normotension to treatable HTN (stages 1 and 2) (*P* < 0.05) in this study. These findings may be helpful in the management of HTN.

MS considerably increases the risk of cardiovascular and renal events in HTN [19] but represents a useful and simple clinical concept that allows for the early detection of DM and CVD [11]. Lifestyle modification is the cornerstone of management in all patients with pre-HTN or with the MS [2]. Elevated levels of WHR, BMI, FPG, TC, and TG were reportedly responsible for the progression of pre-HTN stage to HTN stage1 [17]. Thus, the treatment of the individual components of the syndrome and improvement of modifiable risk factors may be necessary to reduce the triad of obesity, MS, and high BP [19]. 

Pre-HTN is not a disease category but will identify individuals at high risk of developing hypertension, for adequate intervention to prevent or delay the development of the disease. In this present study, 197 (36.9%) of the traders had pre-HTN. 80 (47.1%) of the males and 117 (32.1%) of females had pre-HTN. Only 44 (22.3%) of traders with pre-HTN had MS (Table 1), a finding at variance with a previous report indicating that a vast majority of individuals with pre-HTN had MS [2].

The synergistic impact of HTN and other components of MS illustrate the need for screening for the MS in patients with HTN at initial diagnosis [8]. WHR and WHT may be objective and cheap measures in the assessment of the MS and may further refine cardiovascular risk stratification in HTN. These measures have the advantage of not contaminating the environment and reducing concerns of inaccuracy from mercury and nonmercury sphygmomanometers, respectively [2]. Patients with HTN and MS are at increased risk of CVD and require more vigorous nondrug preventive approach [13]. There is an indication for metabolic screening, dietary and lifestyle modification not only in all patients with HTN at the first diagnosis [8] but also in traders with pre-HTN and the community.

## 4. Conclusion

Metabolic alterations and HTN were observed in traders studied. The significant percentage of individuals with pre-HTN particularly in those without MS is of great concern. Health education and promotion, metabolic screening, and adoption of healthy lifestyles by all persons are critical for the prevention of high BP and should be included in the routine workup and management of patients with HTN.

## Figures and Tables

**Table 1:** Association of BP groups with gender and MS in traders.

Blood pressure classes	Metabolic syndrome		Total	*χ* ^2^	*P*
	Non-MS	MS			
*n* (%)	357 (66.9%)	177 (33.1%)	534 (100%)		
Normotension	133 (93%)	10 (7.0%)	143 (100%)	137.3	<0.001^†^
Pre-HTN	153 (77.7%)	44 (22.3%)	197 (100%)		
Stage 1 HTN	47 (41.2%)	67 (58.8%)	114 (100%)		
Stage 2 HTN	24 (30%)	56 (70%)	80 (100%)		

	Gender				
	Male	Female			

*n* (%)	170 (31.8%)	364 (24.5%)	534 (100%)		
Normotension	54 (31.8%)	89 (24.2%)	143 (26.8%)	25.1	<0.001^†^
Pre-HTN	80 (47.1%)	117 (32.1%)	197 (36.9%)		
Stage 1 HTN	22 (12.9%)	92 (25.3%)	114 (21.3%)		
Stage 2 HTN	14 (8.2%)	66 (18.1%)	80 (15.0%)		

values are in number of participants with percentage in parenthesis, *n*: number of participants, %: percentage, *P*: probability, ^†^: significant, non-MS: non-metabolic syndrome, MS: metabolic syndrome, HTN: hypertension, *χ* ^2^: chi-square test.

**Table 2:** Comparison of components of MS among BP groups.

Variables	BP Group	Value	BP group	Value	*P*
SBP (mmHg)	Normotension	107.4 ± 4.8	Pre-HTN	122.0 ± 6.4	<0.001^†^
	Normotension	107.4 ± 4.8	Stage 1 HTN	139.0 ± 8.6	<0.001^†^
	Normotension	107.4 ± 4.8	Stage 2 HTN	170.1 ± 25.5	<0.001^†^
	Pre-HTN	122.0 ± 6.4	Stage 1 HTN	139.0 ± 8.6	<0.001^†^
	Pre-HTN	122.0 ± 6.4	Stage 2 HTN	170.1 ± 25.5	<0.001^†^
	Stage 1 HTN	139.0 ± 8.6	Stage 2 HTN	170.1 ± 25.5	<0.001^†^

DBP (mmHg)	Normotension	69.4 ± 2.9	Pre-HTN	76.6 ± 5.0	<0.001^†^
	Normotension	69.4 ± 2.9	Stage 1 HTN	85.9 ± 6.2	<0.001^†^
	Normotension	69.4 ± 2.9	Stage 2 HTN	102.5 ± 12.8	<0.001^†^
	Pre-HTN	76.6 ± 5.0	Stage 1 HTN	85.9 ± 6.2	<0.001^†^
	Pre-HTN	76.6 ± 5.0	Stage 2 HTN	102.5 ± 12.8	<0.001^†^
	Stage 1 HTN	85.9 ± 6.2	Stage 2 HTN	102.5 ± 12.8	<0.001^†^

WC (cm)	Normotension	88.2 ± 12.0	Pre-HTN	92.6 ± 13.6	0.002^†^
	Normotension	88.2 ± 12.0	Stage 1 HTN	98.5 ± 12.4	<0.001^†^
	Normotension	88.2 ± 12.0	Stage 2 HTN	102.0 ± 12.7	<0.001^†^
	Pre-HTN	92.6 ± 13.6	Stage 1 HTN	98.5 ± 12.4	<0.001^†^
	Pre-HTN	92.6 ± 13.6	Stage 2 HTN	102.0 ± 12.7	<0.001^†^

FPG (mmol/L)	Normotension	4.7 ± 1.4	Stage 2 HTN	5.0 ± 1.9	0.042^†^
	Stage 1 HTN	4.6 ± 0.9	Stage 2 HTN	5.0 ± 1.9	0.034^†^

TG (mmol/L)	Normotension	0.7 ± 0.3	Stage 1 HTN	0.8 ± 0.4	0.047^†^
	Normotension	0.7 ± 0.3	Stage 2 HTN	0.9 ± 0.4	0.001^†^
	Pre-HTN	0.8 ± 0.4	Stage 2 HTN	0.9 ± 0.4	0.047^†^

*P*: probability, ^†^: significant, values are in mean ± s.d, *n*: number of participants, BP: blood pressure, SBP: systolic blood pressure, DBP: diastolic blood pressure, WC: waist circumference, TG: Triglyceride, FPG: fasting plasma glucose, MS: metabolic syndrome, HTN: hypertension, number of participants in normotension, pre-HTN, stage 1 HTN, stage 2 HTN groups = 143, 197, 114, 80, respectively; total number of participants = 534.

**Table 3:** Comparison of non-MS components among BP groups.

Variable	BP group	Value	BP group	Value	*P*
Height (m)	Normotension	1.64 ± 0.08	Stage 1 HTN	1.61 ± 0.07	0.041^†^
	Normotension	1.64 ± 0.08	Stage 2 HTN	1.61 ± 0.15	0.039^†^
	Pre-HTN	1.65 ± 0.08	Stage 1 HTN	1.61 ± 0.07	0.003^†^
	Pre-HTN	1.65 ± 0.08	Stage 2 HTN	1.61 ± 0.15	0.004^†^

Body weight (kg)	Normotension	65.1 ± 11.4	Pre-HTN	69.6 ± 13.8	0.002^†^
	Normotension	65.1 ± 11.4	Stage 1 HTN	73.1 ± 13.6	<0.001^†^
	Normotension	65.1 ± 11.4	Stage 2 HTN	76.5 ± 15.8	<0.001^†^
	Pre-HTN	69.6 ± 13.8	Stage 1 HTN	73.1 ± 13.6	0.028^†^
	Pre-HTN	69.6 ± 13.8	Stage 2 HTN	76.5 ± 15.8	<0.001^†^

BMI (kg/m^2^)	Normotension	24.4 ± 4.4	Pre-HTN	25.7 ± 5.1	0.024^†^
	Normotension	24.4 ± 4.4	Stage 1 HTN	28.1 ± 5.0	<0.001^†^
	Normotension	24.4 ± 4.4	Stage 2 HTN	29.3 ± 6.3	<0.001^†^
	Pre-HTN	25.7 ± 5.1	Stage 1 HTN	28.1 ± 5.0	<0.001^†^
	Pre-HTN	25.7 ± 5.1	Stage 2 HTN	29.3 ± 6.3	<0.001^†^

HC (cm)	Normotension	97.5 ± 9.9	Pre-HTN	100.7 ± 10.8	<0.006^†^
	Normotension	97.5 ± 9.9	Stage 1 HTN	105.2 ± 10.3	<0.001
	Normotension	97.5 ± 9.9	Stage 2 HTN	106.6 ± 10.6	<0.001^†^
	Pre-HTN	100.7 ± 10.8	Stage 1 HTN	105.2 ± 10.3	<0.001^†^
	Pre-HTN	100.7 ± 10.8	Stage 2 HTN	106.6 ± 10.6	<0.001^†^

PBF	Normotension	28.4 ± 11.9	Stage 1 HTN	37.6 ± 9.9	<0.001^†^
	Normotension	28.4 ± 11.9	Stage 2 HTN	38.8 ± 9.7	<0.001^†^
	Pre-HTN	30.7 ± 11.5	Stage 1 HTN	37.6 ± 9.9	<0.001^†^
	Pre-HTN	30.7 ± 11.5	Stage 2 HTN	38.8 ± 9.7	<0.001^†^

WHT	Normotension	54.1 ± 8.2	Pre-HTN	56.4 ± 8.9	0.036^†^
	Normotension	54.1 ± 8.2	Stage 1 HTN	61.2 ± 8.1	<0.001^†^
	Normotension	54.1 ± 8.2	Stage 2 HTN	64.6 ± 16.8	<0.001^†^
	Pre-HTN	56.4 ± 8.9	Stage 1 HTN	61.2 ± 8.1	<0.001^†^
	Pre-HTN	56.4 ± 8.9	Stage 2 HTN	64.6 ± 16.8	<0.001^†^
	Stage 1 HTN	61.2 ± 8.1	Stage 2 HTN	64.6 ± 16.8	0.021^†^

WHR	Normotension	0.9 ± 0.1	Pre-HTN	0.92 ± 0.1	0.048^†^
	Normotension	0.9 ± 0.1	Stage 1 HTN	0.94 ± 0.1	<0.001
	Normotension	0.9 ± 0.1	Stage 2 HTN	0.96 ± 0.1	<0.001^†^
	Pre-HTN	0.92 ± 0.1	Stage 1 HTN	0.94 ± 0.1	0.030^†^
	Pre-HTN	0.92 ± 0.1	Stage 2 HTN	0.96 ± 0.1	<0.001^†^
	Stage 1 HTN	0.94 ± 0.1	Stage 2 HTN	0.96 ± 0.1	0.030^†^

TC (mmol/L)	Normotension	3.5 ± 1.1	Stage 1 HTN	3.9 ± 1.0	0.016^†^
	Normotension	3.5 ± 1.1	Stage 2 HTN	3.9 ± 1.1	0.018^†^

LDLC (mmol/L)	Normotension	2.1 ± 0.9	Stage 1 HTN	2.4 ± 0.9	0.032^†^
	Normotension	2.1 ± 0.9	Stage 2 HTN	2.4 ± 1.0	0.032^†^

values are in mean ± s.d, *P*: probability, ^†^: significant, HTN: hypertension, MS: metabolic syndrome, PBF: Percentage body fat, BP: blood pressure, WHT: waist to height ratio, WHR: waist to hip ratio, TC: Total Cholesterol, LDLC: low density lipoprotein cholesterol, number of participants in normotension, pre-HTN, Stage 1 HTN, Stage 2 HTN are 143, 197, 114, 80, respectively; total number of participants = 534.
